# Machine learning-based prediction models for accidental hypothermia patients

**DOI:** 10.1186/s40560-021-00525-z

**Published:** 2021-01-09

**Authors:** Yohei Okada, Tasuku Matsuyama, Sachiko Morita, Naoki Ehara, Nobuhiro Miyamae, Takaaki Jo, Yasuyuki Sumida, Nobunaga Okada, Makoto Watanabe, Masahiro Nozawa, Ayumu Tsuruoka, Yoshihiro Fujimoto, Yoshiki Okumura, Tetsuhisa Kitamura, Ryoji Iiduka, Shigeru Ohtsuru

**Affiliations:** 1 Department of Primary Care and Emergency Medicine, Graduate School of Medicine, Kyoto University, ShogoinKawaramachi54, Sakyo, Kyoto, 606-8507 Japan; 2 Preventive Services, School of Public Health, Kyoto University, Kyoto, Japan; 3 Department of Emergency and Critical Care Medicine, Japanese Red Cross Society, Kyoto Daini Hospital, Kyoto, Japan; 4 Department of Emergency Medicine, Kyoto Prefectural University of Medicine, Kyoto, Japan; 5 Senri Critical Care Medical Center, Saiseikai Senri Hospital, Suita, Japan; 6 Department of Emergency, Japanese Red Cross Society, Kyoto Daiichi Red Cross Hospital, Kyoto, Japan; 7 Department of Emergency Medicine, Rakuwa-kai Otowa Hospital, Kyoto, Japan; 8 Department of Emergency Medicine, Uji-Tokushukai Medical Center, Uji, Japan; 9 Department of Emergency Medicine, North Medical Center, Kyoto Prefectural University of Medicine, Kyoto, Japan; 10 Department of Emergency and Critical Care Medicine, National Hospital Organization, Kyoto Medical Center, Kyoto, Japan; 11 Department of Emergency and Critical Care Medicine, Saiseikai Shiga Hospital, Ritto, Japan; 12 Department of Emergency and Critical Care Medicine, Kyoto Min-Iren Chuo Hospital, Kyoto, Japan; 13 Department of Emergency Medicine, Yodogawa Christian Hospital, Osaka, Japan; 14 Department of Emergency Medicine, Fukuchiyama City Hospital, Fukuchiyama, Japan; 15 Division of Environmental Medicine and Population Sciences, Department of Social and Environmental Medicine, Graduate School of Medicine, Osaka University, Osaka, Japan

**Keywords:** Accidental hypothermia, Machine learning, Artificial intelligence, Lasso, Random forest, Gradient boosting tree, Prediction

## Abstract

**Background:**

Accidental hypothermia is a critical condition with high risks of fatal arrhythmia, multiple organ failure, and mortality; however, there is no established model to predict the mortality. The present study aimed to develop and validate machine learning-based models for predicting in-hospital mortality using easily available data at hospital admission among the patients with accidental hypothermia.

**Method:**

This study was secondary analysis of multi-center retrospective cohort study (J-point registry) including patients with accidental hypothermia. Adult patients with body temperature 35.0 °C or less at emergency department were included. Prediction models for in-hospital mortality using machine learning (lasso, random forest, and gradient boosting tree) were made in development cohort from six hospitals, and the predictive performance were assessed in validation cohort from other six hospitals. As a reference, we compared the SOFA score and 5A score.

**Results:**

We included total 532 patients in the development cohort [*N* = 288, six hospitals, in-hospital mortality: 22.0% (64/288)], and the validation cohort [*N* = 244, six hospitals, in-hospital mortality 27.0% (66/244)]. The C-statistics [95% CI] of the models in validation cohorts were as follows: lasso 0.784 [0.717–0.851] , random forest 0.794[0.735–0.853], gradient boosting tree 0.780 [0.714–0.847], SOFA 0.787 [0.722–0.851], and 5A score 0.750[0.681–0.820]. The calibration plot showed that these models were well calibrated to observed in-hospital mortality. Decision curve analysis indicated that these models obtained clinical net-benefit.

**Conclusion:**

This multi-center retrospective cohort study indicated that machine learning-based prediction models could accurately predict in-hospital mortality in validation cohort among the accidental hypothermia patients. These models might be able to support physicians and patient’s decision-making. However, the applicability to clinical settings, and the actual clinical utility is still unclear; thus, further prospective study is warranted to evaluate the clinical usefulness.

**Supplementary Information:**

The online version contains supplementary material available at 10.1186/s40560-021-00525-z.

## Background

Accidental hypothermia is an unintentional decrease in core body temperature below 35 °C with high risks of fatal arrhythmia, multiple organ failure, and mortality (24–40%) [1–4]. Therefore, patients with accidental hypothermia should be immediately evaluated to determine the severity and to consider the treatment strategy. However, accidental hypothermia is relatively rare (approximately 5–10 cases of annual emergency visits in each emergency department) [2]; thus, it is challenging for inexperienced medical staff to accurately estimate the prognosis. Although few prediction models or scales have been suggested earlier to predict mortality [5–8], there is no established model.

Recently, the machine learning technique has been developed and applied to predict the outcome in emergency and critical care settings [9–17]. If machine learning predicts the clinical outcome promptly and it is available in the emergency department using the electronic medical chart along with other applications, it can help to alert the inexperienced medical staff in advance. Further, the predicted probability of the clinical outcome could prove to be essential information for the patients and their family members to decide the invasive treatment strategy. Although few machine learning-based predictions have been validated in emergency and critical care fields [9–20], most of the previous research focused only on frequent emergencies such as triage in the emergency department, trauma, sepsis, or cardiovascular events [9–20]. In contrast, for less frequent emergency conditions such as accidental hypothermia, the validity of machine learning has not yet been studied. Therefore, the present study aimed to develop and validate machine learning-based models for predicting in-hospital mortality using easily available data at hospital admission among patients with accidental hypothermia.

## Methods

### Ethical considerations

This study complied with the Transparent Reporting of a Multivariable Prediction Model for Individual Prognosis or Diagnosis (TRIPOD) statement regarding the reporting of the study’s methods and results [21]. According to the Ethical Guidelines for Medical and Health Research Involving Human Subjects in Japan [22], the ethics committee of the participating center approved the registry protocol and retrospective analysis of de-identified data in this study with a waiver of informed consent, because this study used only anonymized data about already-existing specimens or information. Further, information about the study was made available to the public, and the opportunities to refuse participation in the study were guaranteed (ethical approval ID of representative institution, Kyoto Prefectural University of Medicine: ERB-C-633).

### Study design and settings

This study is a secondary analysis of the multi-center retrospective cohort study (the J-point registry) that included patients with accidental hypothermia. The details of the J-point registry have been previously reported [2, 5, 23–25] and described (see Supplementary Appendix 1 in Additional file 1). In summary, the registry includes patients who were diagnosed and treated for hypothermia in 12 emergency departments in urban areas of Kyoto, Osaka, and Shiga prefectures in Japan between 1 April 2011 and 31 March 2016.

### Study population

This study included all adult patients (≥ 16 years) with a body temperature of 35 °C or lower at admission to the emergency department in the J-point registry. We excluded patients whose body temperature was higher than 35 °C or unknown and with missing fundamental data regarding age, sex, and mortality. We split the included patients into two cohorts based on the geographical location for model development and external validation [26, 27]. The development cohort was created using six emergency departments in Kyoto City, while the validation cohort was created using the other six emergency departments from Shiga, Osaka, and Kyoto prefectures except for Kyoto City. Generally, external validation of prediction models requires different patient profiles. Therefore, this validation cohort was considered appropriate for external validation because the sample splitting was based on geographical location and each cohort was expected to be heterogeneous and consisted of different patient profiles [26, 28].

### Data collection and patient outcomes

We collected the following patient characteristics and clinical information: sex, age, the activity of daily living (ADL) and comorbidities, vital signs at hospital arrival (body temperature, systolic blood pressure, heart rate, and Glasgow Coma Scales) and initial blood gas assessment, blood test results at hospital arrival, sequential organ failure assessment (SOFA) score within 24 h after admission, and rewarming procedures and in-hospital mortality. Details of these variables are provided in Supplementary Appendix 1, Additional file 1. The outcome of interest was in-hospital mortality.

### Variable selection, data preparation, and handling missing data

From the collected data mentioned above, we excluded those variables that were missing for over 30% of the time, and finally, we selected 29 predictor candidates that could be measured at the patient’s hospital arrival. For continuous variables, we treated outliers and obvious contradictory values as missing. For dealing with missing variables, we performed multiple imputations to impute the missing values using the “missForest” package [29, 30]. This imputation technique is a nonparametric algorithm that can accommodate nonlinearities and interactions, and the single point estimates can be generated accurately by a random forest [29, 30]. The advantages of using the random forest model are that it can handle continuous as well as categorical responses, requires very little tuning, and provides an internally cross-validated error estimate [29, 30]. Missingness was imputed using all predictors, outcomes, and other covariates. We did not perform the sample size estimation because of the retrospective nature of the study. There is a consensus on the importance of having an adequate sample size; however, there is no generally accepted approach for estimating the required sample size when developing and validating risk prediction models [28].

### Statistical analyses

#### Patient characteristics and predictors

We described the patients’ characteristics and predictor candidates in each cohort. Continuous variables were described as medians and interquartile ranges (IQRs), while categorical variables were described as numbers and percentages.

#### Machine learning model

Based on previous studies [9–16], we chose the following three machine learning techniques to develop the prediction model in the development cohort: (1) logistic regression with least absolute shrinkage and selection operator (lasso) [9, 14, 15], (2) random forest [9, 15, 16, 31], and (3) gradient-boosting decision tree (gradient boosting tree) [13, 15, 31, 32]. The details of these techniques have been described earlier. As a summary, lasso regularization can choose a few relevant variables and ignore others to reduce the model complexity and prevent overfitting [33–35]. This feature selection can also enable us to interpret the model. For the training, we used 10-fold cross-validation by the “glmnet” package [36] to select the optimal value of the penalty parameter (lambda) and calculated the beta coefficient of the selected variables. Random forest is an ensemble learning method that consists of hundreds or thousands of decision trees [37]. It trains each one on a slightly different set of observations using bootstrapping, and the final predictions are made by averaging the predictions of each individual tree. The gradient boosting tree is another tree-based ensemble learning method similar to a random forest [32]. One of the differences between them is how the trees are built. Random forest trains each tree independently, while gradient boosting trains one tree sequentially based on the previous ones. This additive model works in a forward stage-wise manner, introducing a tree to improve the shortcomings of the existing tree. For developing the random forest and gradient boosting tree models, we performed optimization of the hyperparameters by grid search strategy using the “ranger” and “caret” packages [38, 39]. To understand the contribution of predictors to the models, we showed that the variable importance scaled as the maximum value is 100 [39, 40].

#### Reference model

To compare the predictive performance, we chose the SOFA score and the 5A score as a reference. The SOFA scoring system is the most common severity scale in critical care to evaluate the degree of multiple organ failure, and it was reported to perform well to distinguish the prognosis among the patients with accidental hypothermia admitted to the intensive care unit [41, 42]. We assumed a linear relationship between the SOFA and in-hospital mortality; thus, we considered the SOFA score as a continuous variable and fitted the logistic regression model in the development cohort. The “5A score” was previously developed to predict in-hospital mortality using a logistic regression model with variable selection by clinical experience and validated using the same development and validation cohort in the J-point registry [5]. This model consists of the age, ADL status, hemodynamic status (near arrest), pH, and serum albumin level [5]. The equation of the 5A score used to calculate the probability of in-hospital mortality is described in Supplementary Appendix 2, Additional file 1.

#### Assessment of the performance

For the assessment of predictive performance, developed models were applied to the validation cohort as external validation. The receiver operating curves (ROCs) were drawn, and the C-statistics (also known as areas under the curve) with the 95% confidence interval (95% CI) were calculated as discrimination measures. Further, the C-statistics were compared to the 5A score using the Delong test [43]. For assessment of calibration, calibration plots were drawn using a locally weighted scatter plot smoothing curve to indicate the relationship between the predicted and observed probability of in-hospital mortality in the validation cohort [27]. As an assessment of clinical utility, the net-benefit values of the models were calculated, and the decision curves were shown [44, 45]. The details of the net-benefit and decision curve analysis are explained in Supplementary Appendix 1, Additional file 1. All analyses were performed using the JMP Pro® 14 software (SAS Institute Inc., Cary, NC, USA) and R software (version 1.1.456; R Studio Inc., Boston, MA, USA).

## Results

### Patient characteristics

**Fig. 1:**
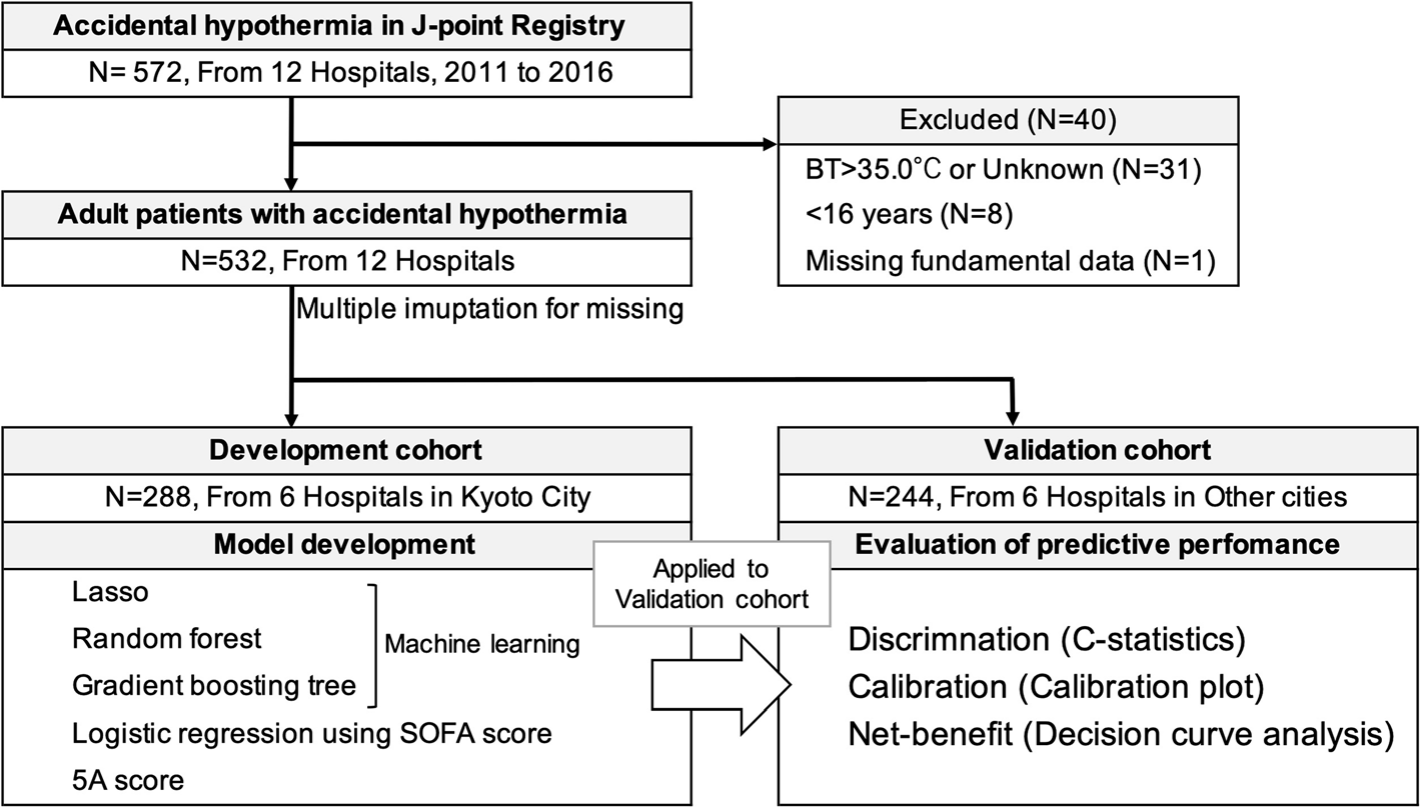
Study flowchart

Among the 572 patients in the J-point registry, 532 patients were ultimately included, and those with missing values data were imputed; finally, the patients were divided into the development cohort [*N* = 288, six hospitals, in-hospital mortality 22.0% (64/288), median age (IQR) 79 (69–87)] and the validation cohort [*N* = 244, six hospitals, in-hospital mortality 27.0% (66/244), median age (IQR) 79 (64–87)]. The study flow chart and other characteristics, and the laboratory data of the patients are shown in Fig. 1, and Tables 1 and 2, respectively. Missing variables are shown in Supplementary Table 1, Additional file 1. The predictor candidates are described by outcomes in Supplementary Table 2, Additional file 1.

**Table 1:** Patients’ characteristics

Variables	Development cohort	Validation cohort
	(*N* = 288)	(*N* = 244)
**Men**	144 (50.0%)	126 (51.6%)
**Age, years**	79 (69–87)	79 (64–87)
< 60	37 (12.8%)	47 (19.3%)
60–69	35 (12.2%)	37 (15.2%)
70–79	75 (26.0%)	48 (19.7%)
≥ 80	140 (48.6%)	117 (48.0%)
**Activities of daily living**		
Disturbance	96 (33.3%)	66 (27.0%)
**Comorbidity**		
Cardiovascular diseases	126 (43.8%)	111 (45.5%)
Neurological diseases	53 (18.4%)	40 (16.4%)
Endocrine diseases	83 (28.8%)	47 (19.3%)
Psychiatric diseases	55 (19.1%)	63 (25.8%)
Malignant diseases	12 (4.2%)	4 (1.6%)
Dementia	57 (19.8%)	51 (20.9%)
Other	56 (19.4%)	38 (15.6%)
**External and minimally invasive rewarming**		
Warm intravenous fluid	223 (77.4%)	168 (68.9%)
Forced warm air	80 (27.8%)	4 (1.6%)
Warm environment, warm blanket	242 (84.0%)	222 (91.0%)
Other	23 (8.0%)	15 (6.1%)
**Active internal rewarming**		
Lavage	29 (10.1%)	15 (6.1%)
CHDF	4 (1.4%)	17 (7.0%)
VV-ECMO	0 (0%)	2 (0.8%)
VA-ECMO	3 (1.0%)	17 (7%)
**In-hospital mortality**	64 (22.2%)	66 (27.0%)

Categorical variables: *n* (%), continuous variables: median [interquartile range]

*CHDF* Continuous hemodiafiltration, *VV-ECMO* Veno-venous extracorporeal membrane oxygenation, *V-A ECMO* Veno-arterial membrane oxygenation

**Table 2:** Vital signs and Laboratory data

Variables	Development cohort	Validation cohort
	(*N* = 288)	(*N* = 244)
Vital signs		
Body temperature	30.7 (28.3–32.6)	31 (28–32.7)
Heart rate	65 (50–82)	63 (45–84)
SBP	116 (93–139)	113 (87–136)
GCS	8 (5–11)	8 (4–11)
13–15	105 (36.5%)	103 (42.2%)
9–12	96 (33.3%)	68 (27.9%)
3–8	87 (30.2%)	73 (29.9%)
Cardiac arrest	5 (1.7%)	16 (6.6%)
Blood gas assessment		
pH	7.32 (7.26–7.36)	7.31 (7.23–7.37)
PaCO2	42.1 (32.8–47.8)	43.8 (37.3–50.4)
PaO2	115.2 (90.1–156)	115.6 (76.3–183.8)
HCO3	21 (15.6–25.4)	21.6 (16.7–25.3)
Base Excess	− 4.3 (− 10.2–0.1)	− 4.4 (− 9.6–0.2)
Lactate	2.6 (1.4–5.1)	3.2 (1.6–6.6)
Blood test results		
WBC	82.1 (53.3–127.3)	83 (51.3–120.8)
Hgb	11.7 (10–13.4)	12 (10.3–13.5)
Hct	35.3 (30–40.3)	36.4 (32–40.7)
PLT	17.1 (12.2–22.8)	19.4 (13.5–24.5)
Glu	127.5 (88.8–178)	141.7 (101–195)
Na	139 (135–143)	140 (137–143)
K	4.2 (3.6–4.7)	4 (3.5–4.6)
Cl	103 (99–107)	103 (100–107)
Ca	8.8 (8.4–9.3)	8.8 (8.3–9.2)
Cr	1.1 (0.6–2)	0.9 (0.6–1.6)
BUN	38 (20.4–60)	28.2 (17–51.7)
TP	6.5 (5.8–7)	6.4 (5.7–7.2)
Alb	3.4 (2.9–3.9)	3.5 (3–4)
T-bil	0.6 (0.5–1.1)	0.6 (0.4–0.9)
CK	503 (142.3–1388)	418.5 (129–1281.5)
CRP	1.8 (0.4–6.2)	1.1 (0.1–4)
Score		
SOFA	4 [3–6]	4 [2–7]
5A score	4 [3–5]	4 [2–5]

*ADL* Activity of daily living, *BT* Body temperature, *SBP* Systolic blood pressure, *GCS* Glasgow coma scale, *WBC* White blood cell count, *Hgb* Hemoglobin, *Hct* Hematocrit, *PLT* Platelet count, *BUN* Blood urea nitrogen, *TP* Total protein, *Alb* Serum albumin, *T-bil* Total bilirubin, *CK* Creatine kinase, *SOFA* Sequential organ failure assessment score, categorical variables: *n* (%), continuous variables: median [interquartile range]

### Model development

**Fig. 2:**
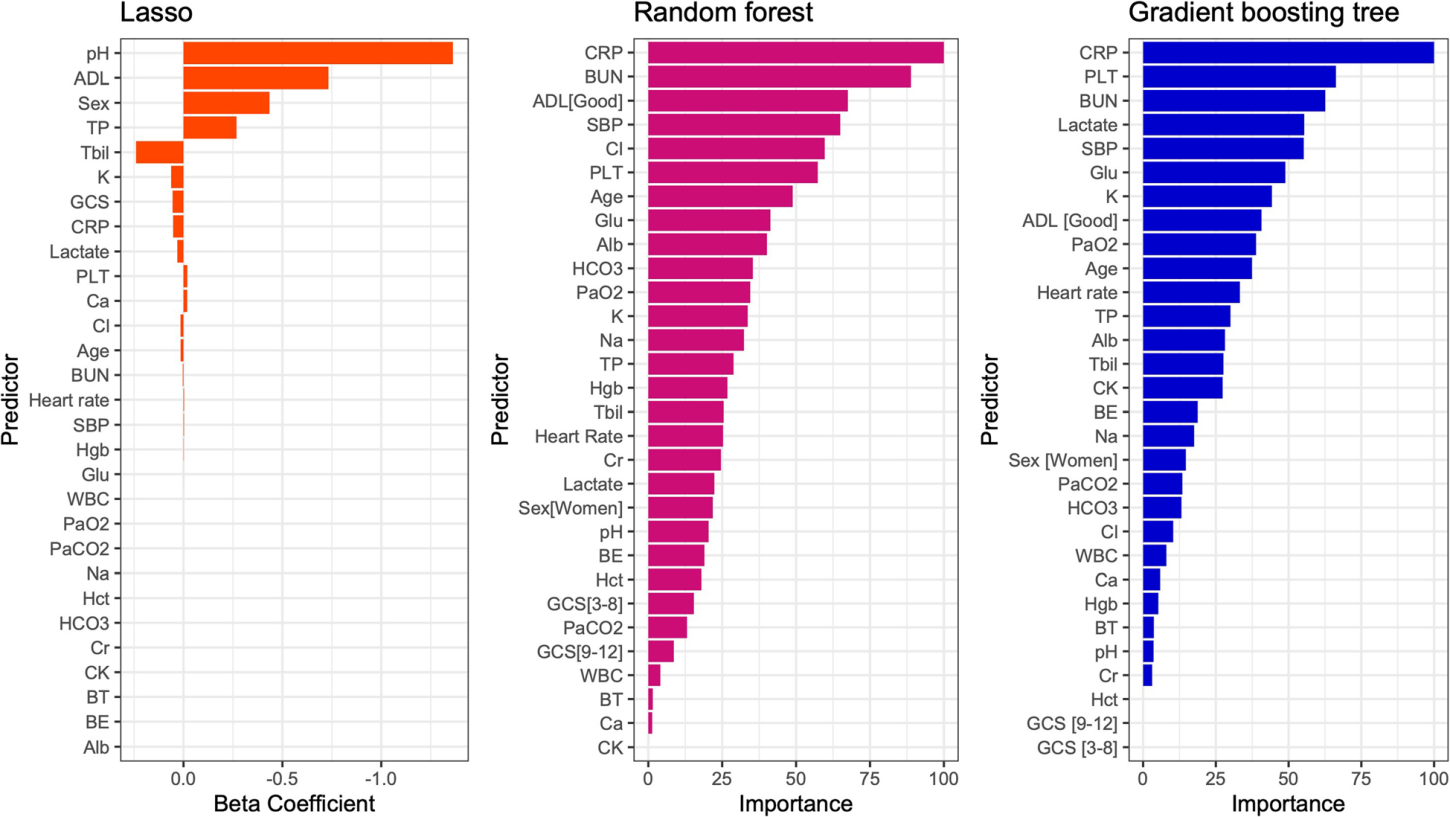
The features of the models. Beta coefficients value in lasso and importance of variables in random forest and gradient boosting tree were shown. ADL: activity of daily living, BT: body temperature, SBP: systolic blood pressure, GCS: Glasgow coma scale, WBC: white blood cell count, Hgb: hemoglobin, Hct: hematocrit, PLT: platelet count, BUN: blood urea nitrogen, TP: Total protein, Alb: serum albumin, T-bil: Total bilirubin, CK: creatine kinase

In the final lasso model with the optimal lambda to minimize the mean squared error, 18 selected variables and beta coefficient values are shown in Fig. 2. In the random forest model and gradient boosting tree model, the importance of the predictors is also indicated in Fig. 2. The other hyperparameters of machine learning model are described in Supplementary Table 3, Additional file 1. Based on the distribution of outcome by SOFA score in the development cohort, it was reasonable to assume the association between SOFA score and in-hospital mortality as a linear relationship (Supplementary Fig. 1, Additional file 1). The logistic regression model using the SOFA score showed that the beta-coefficient value was 0.300 for each point of the SOFA score, and the intercept was − 2.847. For the 5A score, we used the previously developed model described in Supplementary Appendix 3, Additional file 1.

### Model performance in validation cohorts

**Fig. 3:**
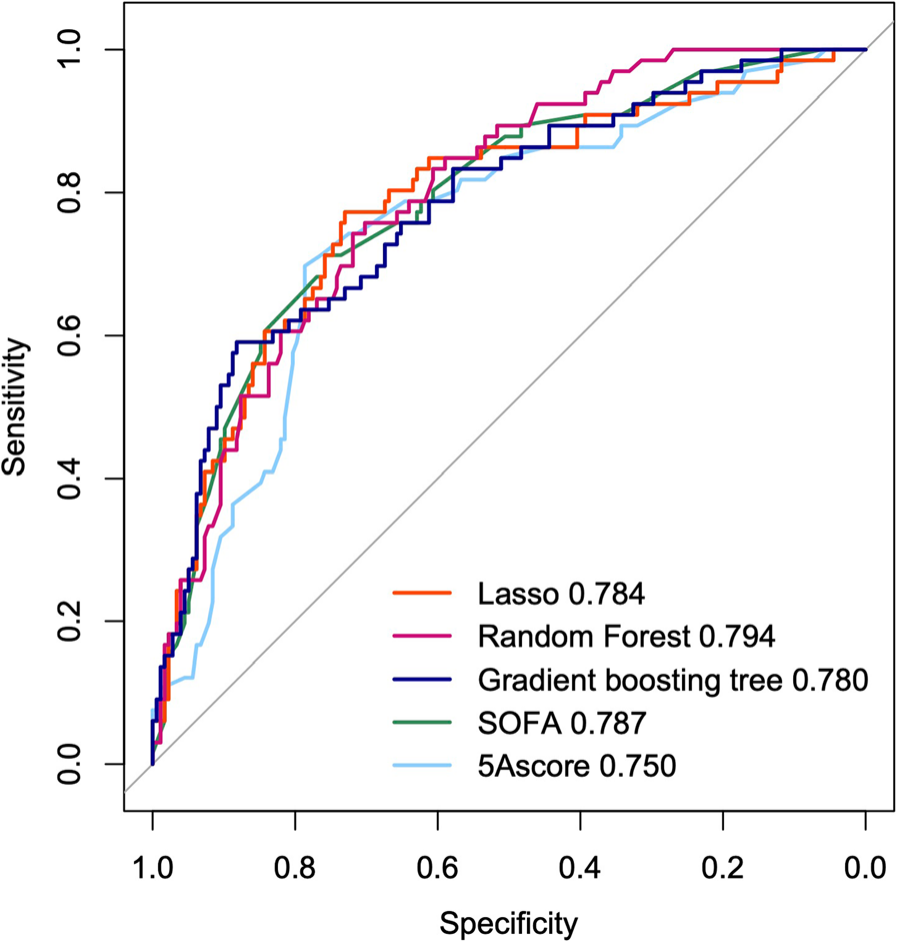
ROC of the models. The C-statistics [95% CI] of the models; lasso: 0.784 [0.717–0.851], random forest: 0.794 [0.735–0.853], boosting tree: 0.780 [0.714–0.847], SOFA: 0.787 [0.722–0.851], and 5A score: 0.750 [0.681–0.820]. CI: confidence interval, ROC: receiver operating curve

**Fig. 4:**
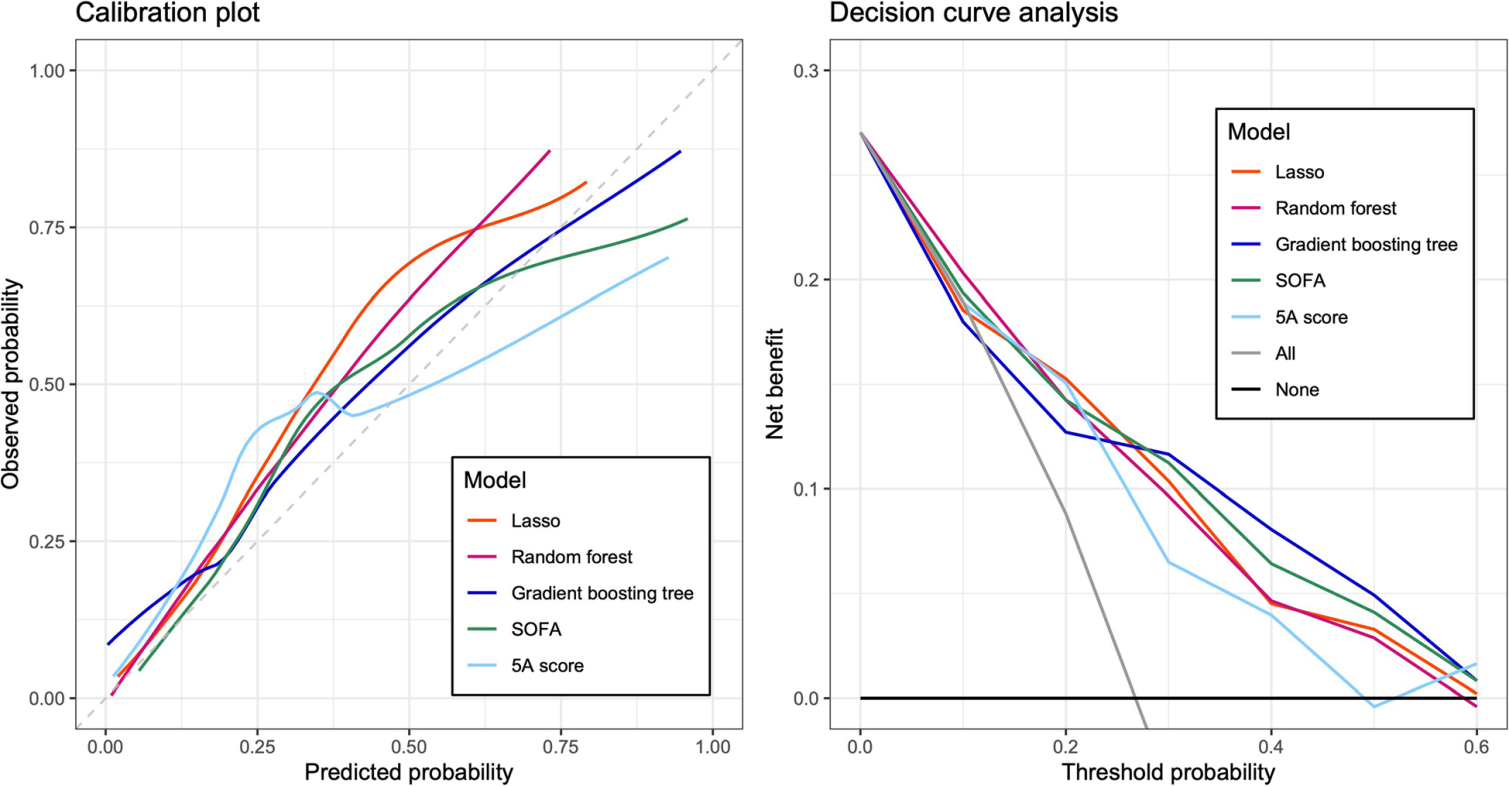
Calibration plot and decision curve analysis. Left: calibration plot, right: decision curve analysis. Calibration plot, *X*-axis: predicted probability, *Y*-axis: observed frequency in validation cohort. Decision curve analysis, X-axis: threshold probability, y-axis: net-benefit. The detail of net-benefit and decision curve analysis is described in Supplementary Appendix 3 in Additional file 1

For discrimination, the C-statistics [95% CI] of the models in validation cohorts were as follows: lasso, 0.784 [0.717-0.851]; random forest, 0.794 [0.735–0.853]; boosting tree, 0.780 [0.714–0.847]; SOFA, 0.787 [0.722–0.851]; and 5A score, 0.750 [0.681–0.820]. The ROCs were plotted in Fig. 3. There was no significant difference in C-statistics compared with the 5A score (see Supplementary Table 4, Additional file 1). For the visual assessment of the calibration plot in the validation cohort (Fig. 4), the boosting tree model and SOFA were well calibrated to the observed overall range of the predicted in-hospital mortality. Although the other models were also calibrated to some extent, the lasso and random forest models were slightly underestimated, and the 5A model was partially over- and underestimated in the range of high predicted in-hospital mortality. In the decision curve analysis, the net-benefit values of the models were higher than the all treatment and none strategy (Fig. 4). Although the net-benefit values of the models were almost the same, the net-benefit of the gradient boosting tree was slightly higher and that of the 5A score was slightly lower than the others.

## Discussion

### Key observation

This multi-center retrospective cohort study indicated that machine learning using the lasso, random forest, and gradient boosting tree had adequate discrimination and calibration performance in predicting in-hospital mortality among patients with accidental hypothermia. Further decision curve analysis showed the net-benefit can be obtained using these prediction models. These results suggested the potential clinical usefulness of these predictions.

### Strength of this study

This study has some strengths compared with previous studies. First, this was the first study to indicate the machine learning-based prediction models for accidental hypothermia, which were validated with adequate discrimination and calibration performance using the external validation cohort. Previously, some prediction models were developed for patients with accidental hypothermia [5–8]; however, to the best of our knowledge, no study has been conducted for the machine learning model. Machine learning has potential advantages in variable selection and modeling in terms of considering high-order interactions between the predictors and nonlinear relationships with the outcome [37, 46]. Therefore, machine learning-based prediction is expected to predict the outcome more accurately. In our study, machine learning-based predictions performed at par with or better than a simple scoring system such as the 5A score in terms of calibration and net-benefit. Therefore, this study indicated that machine learning-based prediction may potentially contribute to better prediction and decision-making.

Second, this study specifically focused on accidental hypothermia, which is a relatively less common situation for investigating the utility of machine learning-based prediction. Due to the lack of an adequate number of severe cases in some institutions, it may be difficult for inexperienced clinicians to accurately predict the prognosis. Meanwhile, some previous studies using machine learning focused on more common situations such as triage for emergency conditions, sepsis, and trauma [9–20]. However, a number of risk stratification systems have been well established for such cases (e.g., SOFA score or quick SOFA score for sepsis [42, 47], Canadian emergency department triage and acuity scale for triage [CTAS] in the emergency department [48], acute physiology and chronic health evaluation 2 [APACHE2] score for critically ill patients [49], or revised trauma score for severe trauma) [50]. Therefore, even if the machine learning system does not work, clinicians can use alternative classic tools in the initial assessment of severity. However, for accidental hypothermia, there are no commonly used models validated with external data. Historically, the Swiss staging system based on the body temperature are used for triage; however, the discrimination performance was reported to be inadequate [5]. Therefore, machine learning that is adapted to patients with relatively less common conditions such as accidental hypothermia may fit the requirement in clinical settings.

Third, we highlighted that machine learning models in this study were built based on the objective information that is available easily and immediately in any emergency department. In some of the previous studies, predictor candidates were selected based on subjective information such as patient’s complaint or information that was inaccurate or unavailable at emergency department admission [9, 13, 14, 17] Prediction models based on less certain or unavailable information might have disadvantages concerning their applicability to other settings. On the other hand, prediction models in this study were mainly built by using objective information such as blood test results. Therefore, this study may be expected to be highly applicable to other settings.

### Interpretation and clinical implication

We suggest some explanations for the potential advantages of the good predictive performance of machine learning models that we have shown in this study. First, machine learning approaches can incorporate the nonlinear interactions between predictors, which cannot be addressed by using traditional modeling [37, 46]. In contrast, the traditional logistic regression model is not suitable to deal with unknown interactions and nonlinear relationships [37, 46]. Second, this modeling study was performed to minimize potential overfitting. Generally, the prediction models developed from the data with a limited number of outcome events are prone to overfitting, and predictive performance may be worse in the external validation dataset [35]. To deal with this limitation, we adapted the cross-validation or bootstrap procedures to reduce the overfitting [37, 46]. Further, we used the ensemble method which is obtained by combining multiple learning algorithms such as random forest or gradient boosting tree, and obtained the flexibility to avoid overfitting [37, 46]. These may contribute to good predictive performance even if the dataset was small. On the other hand, some previous studies reported that the predictive performance of machine learning techniques was not superior to that of the traditional logistic regression model [51–53]. Similar to earlier studies, this study did not show that the machine learning-based model was much better than the 5A score or SOFA model based on the logistic model. However, we believe that these machine learning methods are advantageous especially when background knowledge of the clinical question is lacking. It is because background knowledge or clinical experience is necessary to choose optimal predictors in the logistic model from among many predictor candidates [27]. The 5A score was developed based on background knowledge and clinical experience, and the SOFA score is a well-established scale to assess multiple organ failure. We believe that a machine learning-based model may be convenient for predicting the outcome in the case of accidental hypothermia, in which the number of studies investigating the risk factors or predictive factors is limited.

The clinical implication of this study is that the machine learning-based prediction model would play an important role as an accurate early warning system and convey valuable information that is needed to consider the treatment strategy. If these algorithms are implemented in the electronic medical record system, it can enable clinicians to identify the possibility of in-hospital mortality and to manage the patients appropriately. Further, the actual number of probabilities of in-hospital mortality may be informative to the patients and family members. Especially, most of the patients with accidental hypothermia in urban settings were elderly, and some of them might even withdraw the invasive treatment if they are informed of a high probability of in-hospital mortality. Hence, this study may support machine learning implementation in actual clinical settings. However, some obstacles arise when introducing these techniques in clinical settings. The algorithm of machine learning is so complicated that it is termed a “black box,” and it is not easy to interpret how the probability is calculated. Thus, implementation in clinical settings requires certain software or application. Further, to enable the use of machine learning techniques in a timely manner, a standardized format to extract clinical data would be essential. Although some systems have been used to collect data structurally in the emergency and critical medicine fields, they are not normally dedicated for use in such fields in most institutions in Japan [54, 55]. Therefore, when ease and speed of prediction without special software are considered, traditional prediction models such as the 5A score or SOFA score may be valuable. A possibility could be that machine learning is not superior to traditional prediction in some situations; however, if it is used flexibly and combined with the traditional prediction model, it may prove to be valuable in most clinical settings.

## Limitations

This study has some limitations. First, we attempted to include all the patients with hypothermia admitted to the emergency department using diagnosis coding; however, we might have missed some of the patients who were not coded as hypothermia. This may result in a risk of selection bias. Second, because of the retrospective nature of the data collection by chart review, the validity of the variables and measurement was unclear. For example, the blood test was defined as “initial blood test at hospital arrival”; however, the exact timing was unclear. Further, some variables were missing. For example, saturation was not recorded in the registry, and respiratory rate was not measured in many cases. Although we double-checked the data validity and imputed missing values using rigorous multiple imputation techniques [30], this process may lead to a measurement bias. Third, the exact cause of death in most cases was unclear, because this study did not collect information about autopsy or whether autopsy was performed. Therefore, caution is necessary when interpreting this result. Fourth, the sample size and the number of events were limited, as accidental hypothermia is generally relatively rare. This study has the largest database of information on accidental hypothermia in urban settings; however, the sample size was relatively small. This may cause overfitting of the models and decrease the generalizability of the findings. Finally, the applicability of the model to clinical settings and the actual clinical utility remain unclear. Most clinicians may hesitate to believe that machine learning-based prediction using factors that are not clinically relevant is valuable in clinical decision-making, and we agree to that. Further, we understand that they may prefer to use commonly accepted prediction methods such as the SOFA score even if the performance is the same as that of new techniques. It should be noted that clinical utilization of machine learning techniques is still in a process of development, and a discussion about its clinical utility compared to the traditional way would be necessary. We hope that this study would trigger discussions about the implementation of machine learning-based prediction in the emergency or critical care field. Therefore, further prospective studies would be necessary to overcome these limitations and to identify the generalizability and usefulness of the models in clinical settings.

## Conclusions

This multi-center retrospective cohort study indicates that the prediction model using machine learning can accurately predict in-hospital mortality in the validation cohort in accidental hypothermia patients. The application of these models to actual clinical settings could support physicians’ and patients’ decision-making. However, their applicability to clinical settings and their actual clinical utility remain unclear and warrant further prospective studies.

## Supplementary Information

**Additional file 1: Supplementary Appendix 1**. Explanation of J-point registry. **Supplementary Appendix 2**. Explanation of 5A score. **Supplementary Appendix 3**. Net-benefit and decision curve analysis. **Supplementary Figure 1**. Mortality by SOFA score. **Supplementary Table 1**. Missing value. **Supplementary Table 2**. Predictors described by outcome. **Supplementary Table 3**. Hyperparameters in machine learning models. **Supplementary Table 4**. Difference of C-statistics in each model

## Data Availability

Data sharing is not applicable to this article as the ethics committee has not approved it.

## Abbreviations

ADL: Activity of daily living
CI: Confidence interval
IQR: Interquartile range
SOFA: Sequential organ failure assessment
