# Supplementary material for: Machine learning-based prediction models for accidental hypothermia patients
Source: J Intensive Care. 2021 Jan 9;9:6. doi: 10.1186/s40560-021-00525-z (PMC7797142; doi:10.1186/s40560-021-00525-z)
Supplement: Supplementary file 1 — Additional file 1: Supplementary Appendix 1. Explanation of J-point registry. Supplementary Appendix 2. Explanation of 5A score. Supplementary Appendix 3. Net-benefit and decision curve analysis. Supplementary Figure 1. Mortality by SOFA score. Supplementary Table 1. Missing value. Supplementary Table 2. Predictors described by outcome. Supplementary Table 3. Hyperparameters in machine learning models. Supplementary Table 4. Difference of C-statistics in each model [file 40560_2021_525_MOESM1_ESM.docx]

**Additional file**

**Title:** Machine Learning-Based Prediction models for Accidental Hypothermia patients

**Authors**

Yohei Okada, MD^1,2*^; Tasuku Matsuyama, MD, PhD^3^; Sachiko Morita, MD^4^; Naoki Ehara, MD^5^; Nobuhiro Miyamae, MD^6^; Takaaki Jo, MD^7^; Yasuyuki Sumida, MD^8^; Nobunaga Okada, MD^3,9^; Makoto Watanabe, MD^3^; Masahiro Nozawa, MD^10^; Ayumu Tsuruoka, MD^11^; Yoshihiro Fujimoto, MD^12^; Yoshiki Okumura, MD^13^; Tetsuhisa Kitamura, MD, PhD^14^; Ryoji Iiduka, MD^2^; Shigeru Ohtsuru, MD, PhD^1^

**Contents**

| **Supplementary Appendix 1.** | **Explanation of J-point registry** |
| --- | --- |
| **Supplementary Appendix 2.** | **Explanation of 5A score** |
| **Supplementary Appendix 3.** | **Net-benefit and decision curve analysis** |
| **Supplementary Figure 1.** | **Mortality by SOFA score** |
| **Supplementary Table 1.** | **Missing value** |
| **Supplementary Table 2.** | **Predictors described by outcome** |
| **Supplementary Table 3.** | **Hyperparameters in machine learning models** |
| **Supplementary Table 4.** | **Difference of C-statistics in each model** |

**Supplementary Appendix 1. Explanation of the J-point registry**

We described additional explanation of the J-point registry and other data based on previous literatures. ^1^

**Study design and Setting**

Japanese accidental hypothermia network registry (J-Point registry) was a retrospective cohort study in which the patients with temperature ≤35°C admitted to emergency departments were registered in Japan.^1^ It consists of eight tertiary critical care medical centers (CCMCs) and four non-CCMCs in urban area of Kyoto, Osaka and Shiga Prefectures in Japan. Median and interquartile range of annual ED visit volume in the institutions were 19,651 (Interquartile range 13,281–27,554).^1^ In Japan, CCMCs are certified by the Ministry of Health, Labour and Welfare based on EDs that treat patients for shock, trauma, resuscitation, and critical care which serve approximately 500,000 residents in each region; in these CCMSs, advanced treatment like extracorporeal membrane oxygenation (ECMO) is generally available. ^2^. The non-CCMC centers are public or private hospitals that cover a smaller regional community, and, generally, advanced treatment such as ECMO is unavailable.^3^

**Study population**

This registry included the patients who were diagnosed during the study period, 1 April 2011 to 31 March 2016 using the International Classification of Diseases, Tenth Revision (ICD-10) code T68: ‘Hypothermia’. We excluded those who aged <18 years, did not visit a participating ED, whose body temperature was unknown or >35°C, or where the patient or their family members had refused to be part of the registry.^1^

**Data collection and quality control**

In-hospital data were collected using a predefined uniform data sheet by emergency physicians who were trained in appropriate data extraction during face-to-face or web meetings. The collected data were double-checked by the J-Point registry working group members and confirmed. If an issue was identified, it was returned to the institution for clarification before being entered into the registry.^1^

**Patient data**

The following baseline patient information was collected: sex, age, activities of daily living (ADL) before the accidental hypothermia (independent, needing assistance), past medical history (cardiovascular disease (ischemic heart disease, heart failure, arrhythmia, hypertension, other), neurological disease (stroke, epilepsy, Parkinson disease or syndrome, other), endocrine disease (diabetic mellitus, thyroid diseases, adrenal insufficiency, other), psychiatric disease (chronic alcoholic, depression, schizophrenia, other), malignant disease, dementia, other) and mode of arrival at emergency department (walk-in, ambulance).^1^ Activities of daily living (ADL) was defined as daily activity including eating, dressing, getting into or out of a bed or chair, taking a bath or shower and using toilet independently. Disturbance is assessed as the requirement of partial or total assistance for these activities before the accidental hypothermia event, based on the judgement of the physicians or nurses in charge or their families.

**In-hospital data**

Following in-hospital data were collected; vital signs on arrival at hospital (body temperature, blood pressure, heart rate, Glasgow coma scale score), initial blood gas assessment data [pH, PaCO_2_, PaO_2_, HCO_3_^-^, lactate, base excess] and initial laboratory data [white blood cell count (WBC), hemoglobin (Hb), hematocrit (Ht), platelet count (Plt), total protein (TP), albumin (Alb), C-reactive protein (CRP), total bilirubin (T-bil), creatine kinase (CK), blood urea nitrogen (BUN), creatinine (Cr), glucose(Glu), Na^+^, Cl^-^, K^+^, Ca^2+^,], sequential organ failure assessment (SOFA) score, rewarming methods and in-hospital mortality.^1^ SOFA score was calculated for the patients admitted to the intensive care unit (ICU) based on the data within 24 hours after hospital admission. Rewarming procedures were divided into active external/minimally invasive rewarming (warm intravenous fluids, warm blanket, forced warm air, heating pads, warm bath) and active internal rewarming [lavage (stomach, chest, bladder), intravascular warming catheter, hemodialysis, and extracorporeal rewarming using extracorporeal membrane oxygenation].^1^ The blood gas assessment and other laboratory data was measured by the blood sample initially obtained on the arrival at the emergency department. Either atrial or venous blood sample were allowable. It is because pH on the blood gas assessment is known as well correlation between venous and atrial blood sample, and the venous blood sample can be thought as reasonable substitute other than PaO_2_.^4^ Cardiac arrest was defined as heart rate and blood pressure was 0. An outlier was considered as any value more than 3 times the interquantile range from the 10% and 90% quantiles. This method is a default setting in the statistical software JMP Pro® 14 software (SAS Institute Inc., Cary, NC, USA) and is conservatively based on Tukey’s fence method, which is traditionally accepted. ^5^

**Supplementary Appendix 2. Explanation of 5A score**

The development and validation of 5A score is reported by previous literature.^6^ In summary, this score was developed by logistic regression model in the development cohort consist of 6 hospitals in Kyoto city of J-point registry, and validated in the cohort consist of 6 hospitals in other cities. The validation cohort in this study was not involved in developing this model.

Predicted in-hospital mortality: p= 1/{1+Exp-(ax+b)}

ax+b=- 4.2338

+ Age (60-69 years) * 0.675 + Age (70-79 years) * 1.376 + Age (≥80 y.o) * 1.976

+ ADL (distrubance) * 0.918

+ SBP (61-90mmHg) * (-0.146) + near-Arrest * 1.716

+ pH (7.2-7.35) * 0.786 + pH (<7.2) * 1.217

+ Alb (≤3 mg/dl) * 0.952

ADL:Activity of daily living, SBP: systolic blood pressure

near-Arrest: systolic blood pressure ≤ 60 mmHg, unmeasurable, and cardiac arrest,

Alb: serum Albumin

*Original euqation contains coefficient of “unknown” category; however, we omit it.


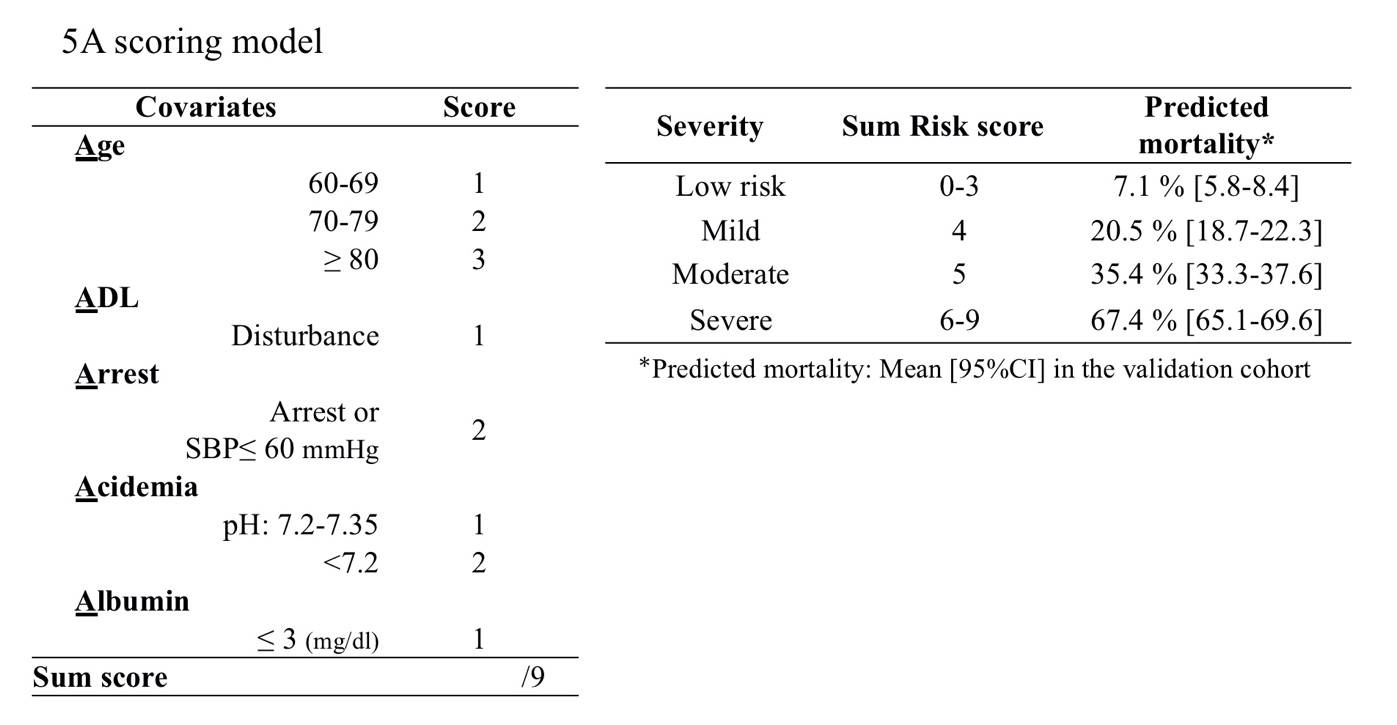
We summarize the model to simple scoring system (5A score) as below.

**Supplementary Appendix 3. Net-benefit and decision curve analysis**

Net-benefit refers to the difference between benefit and weighted harm of the test calculated as:

Net-benefit = proportion of TP − proportion of FP × weighting

Weighting = p / (1 − p)

where, p is threshold probability. Net-benefit refers to the number of FP patients that have clinical importance equal to one TP patient; threshold probability refers to the level of diagnostic certainty above which the patient would be treated based on hospital policy or own preference. For example, if “p = 0.1,” the weighting = 0.1 / (1 − 0.1) = 1/9, meaning that 9 FP is equal to 1 TP, so if 10% of patients are TP (9 FP and 1 TP), all patients should be treated. In general, in decision curve analysis, net-benefit is plotted using index test or prediction under several thresholds of probability. Further, net-benefit is plotted if all patients are treated as positive or negative regardless of the index test result or prediction. Decision curve analysis can help obtain the highest net-benefit. Further reference is available in some previous literatures.^7-9^

In this study, we either assumed all patients to be positive and performed intensive care (All treatment strategy) or considered all patients to be negative and performed no intensive treatment.

**Supplementary Figure 1. Mortality by SOFA score in development cohort**

Bar-plot shows mortality in development cohort by SOFA score.

| **SOFA score** | **Mortality** |
| --- | --- |
| 0-1 | 3.7% (1/27) |
| 2-3 | 11.1% (8/72) |
| 4-5 | 21.1% (19/90) |
| 6-7 | 28.1% (16/57) |
| 8-9 | 36.0% (9/25) |
| 10- | 64.7% (11/17) |

**Supplementary Table 1. Missing value**

|  | **Total cohort** | | **Development cohort** | | **Validation cohort** | |
| --- | --- | --- | --- | --- | --- | --- |
|  | N=532 | | N=288 | | N=244 | |
| Sex | 0 | 0.0% | 0 | 0.0% | 0 | 0.0% |
| Age | 0 | 0.0% | 0 | 0.0% | 0 | 0.0% |
| ADL | 2 | 0.4% | 2 | 0.7% | 0 | 0.0% |
| BT | 0 | 0.0% | 0 | 0.0% | 0 | 0.0% |
| Heart rate | 4 | 0.8% | 2 | 0.7% | 2 | 0.8% |
| SBP | 36 | 6.8% | 15 | 5.2% | 21 | 8.6% |
| GCS | 66 | 12.4% | 32 | 11.1% | 34 | 13.9% |
| pH | 67 | 12.6% | 43 | 14.9% | 24 | 9.8% |
| PaCO2 | 66 | 12.4% | 42 | 14.6% | 24 | 9.8% |
| PaO2 | 79 | 14.8% | 55 | 19.1% | 24 | 9.8% |
| HCO3 | 76 | 14.3% | 48 | 16.7% | 28 | 11.5% |
| BE | 112 | 21.1% | 86 | 29.9% | 26 | 10.7% |
| Lactate | 105 | 19.7% | 65 | 22.6% | 40 | 16.4% |
| WBC | 6 | 1.1% | 1 | 0.3% | 5 | 2.0% |
| Hgb | 5 | 0.9% | 1 | 0.3% | 4 | 1.6% |
| Hct | 7 | 1.3% | 1 | 0.3% | 6 | 2.5% |
| PLT | 7 | 1.3% | 1 | 0.3% | 6 | 2.5% |
| Glu | 47 | 8.8% | 21 | 7.3% | 26 | 10.7% |
| Na | 9 | 1.7% | 1 | 0.3% | 8 | 3.3% |
| K | 8 | 1.5% | 1 | 0.3% | 7 | 2.9% |
| Cl | 15 | 2.8% | 4 | 1.4% | 11 | 4.5% |
| Ca | 61 | 11.5% | 24 | 8.3% | 37 | 15.2% |
| Cr | 7 | 1.3% | 2 | 0.7% | 5 | 2.0% |
| BUN | 8 | 1.5% | 1 | 0.3% | 7 | 2.9% |
| TP | 37 | 7.0% | 15 | 5.2% | 22 | 9.0% |
| Alb | 89 | 16.7% | 48 | 16.7% | 41 | 16.8% |
| T-bil | 11 | 2.1% | 2 | 0.7% | 9 | 3.7% |
| CK | 16 | 3.0% | 4 | 1.4% | 12 | 4.9% |
| CRP | 8 | 1.5% | 2 | 0.7% | 6 | 2.5% |
| SOFA | 117 | 22.0% | 66 | 22.9% | 51 | 20.9% |

ADL: Activity of daily living, BT: Body temperature, SBP: Systolic blood pressure, GCS: Glasgow coma scale, WBC: white blood cell count, Hgb: hemoglobin, Hct: hematocrit, PLT: platelet count, BUN: Blood urea nitrogen, TP: Total protein, Alb: serum albumin, T-bil: Total bilirubin, CK: creatine kinase, SOFA: sequential organ failure assessment score

**Supplementary Table 2. Predictors described by outcome**

| **Variables** | **Development cohort** | | **Validation cohort** | |
| --- | --- | --- | --- | --- |
|  | **Survival** | **Mortality** | **Survival** | **Mortality** |
| Men | 103 (46%) | 41 (18.3%) | 94 (42%) | 32 (14.3%) |
| Age | 78 [66 - 87] | 83 [76 - 88] | 77 [62 - 86] | 82 [68 - 88] |
| ADL: Good | 160 (71.4%) | 32 (14.3%) | 139 (62.1%) | 39 (17.4%) |
| ADL: Disturbance | 64 (28.6%) | 32 (14.3%) | 39 (17.4%) | 27 (12.1%) |
| BT | 30.8 [28.4 - 32.8] | 30.2 [28 - 32.2] | 31.2 [28.3 - 32.8] | 30.5 [26.5 - 32.2] |
| Heart Rate | 67 [51.3 - 82] | 61.5 [42 - 78.3] | 69 [49.8 - 88.3] | 49.5 [29.8 - 72] |
| SBP | 121 [97 - 141] | 99.5 [82.5 - 122.5] | 120 [92 - 138] | 93 [62.3 - 124.7] |
| GCS | 11 [8-14] | 10 [6 - 13] | 13 [9 -14] | 8 [3 - 11] |
| 3-8 | 64 (28.6%) | 23 (10.3%) | 37 (16.5%) | 36 (16.1%) |
| 9-12 | 71 (31.7%) | 25 (11.2%) | 52 (23.2%) | 16 (7.1%) |
| 13-15 | 89 (39.7%) | 16 (7.1%) | 89 (39.7%) | 14 (6.3%) |
| pH | 7.3 [7.3 - 7.4] | 7.3 [7.2 - 7.3] | 7.3 [7.3 - 7.4] | 7.2 [7 - 7.3] |
| PaCO2 | 42.4 [34 - 48.5] | 41.3 [30.4 - 46.2] | 43.7 [36.7 - 48.7] | 44.3 [39 - 60.1] |
| PaO2 | 113 [90 - 148] | 123 [86 - 183] | 116 [82 - 169] | 116 [58 - 202] |
| HCO3 | 21.9 [16 - 25.7] | 18 [12.6 - 24.6] | 22.6 [17.8 - 25.5] | 19.1 [12.5 - 22.8] |
| BE | -3.9 [-9.5 - 0.2] | -6.9 [-12.8 - -1.2] | -3.2 [-8.4 - 0.2] | -9.2 [-19 - -1.7] |
| Lactate | 2.5 [1.4 - 4.7] | 3.1 [1.6 - 7.6] | 2.7 [1.4 - 5] | 5 [1.9 - 9.3] |
| WBC | 83 [54 - 128] | 74.4 [49.5 - 116.9] | 83.5 [55.3 - 117.3] | 80 [45.7 - 134.3] |
| Hgb | 11.9 [10 - 13.4] | 11.1 [9.5 - 13.1] | 12.2 [10.7 - 13.4] | 11.5 [10 - 14] |
| Hct | 35.8 [30.1 - 40.7] | 33.8 [29.6 - 38.8] | 36.4 [32.3 - 40.5] | 36.2 [30.7 - 42.5] |
| PLT | 17.9 [12.9 - 23.6] | 13.9 [9.2 - 19.4] | 20.1 [14 - 26] | 17.1 [12 - 21.5] |
| Glu | 132.5 [97.8 - 182] | 104 [63.8 - 149.8] | 133.4 [101 - 183.3] | 156 [101.5 - 226.8] |
| Na | 139 [135 - 143] | 140 [134 - 144.8] | 140 [137 - 143] | 141 [135 - 143] |
| K | 4 [3.6 - 4.6] | 4.5 [3.8 - 5.2] | 3.9 [3.5 - 4.4] | 4.5 [3.5 - 5.6] |
| Cl | 103 [98.3 - 106] | 104 [100.3 - 112.5] | 103.1 [100 - 107] | 103 [98 - 106.8] |
| Ca | 8.9 [8.4 - 9.3] | 8.6 [8 - 9.2] | 8.9 [8.5 - 9.2] | 8.4 [8 - 9] |
| Cr | 1 [0.6 - 1.7] | 1.6 [0.8 - 2.9] | 0.8 [0.6 - 1.3] | 1.3 [0.8 - 2.5] |
| BUN | 33.9 [19.1 - 52.6] | 53 [31.9 - 82.8] | 24.1 [16.5 - 45] | 41.4 [22.7 - 65.7] |
| TP | 6.6 [6 - 7.3] | 6 [5.5 - 6.7] | 6.6 [5.9 - 7.3] | 5.9 [5.3 - 6.7] |
| Alb | 3.5 [3 - 4] | 3 [2.7 - 3.4] | 3.6 [3.1 - 4] | 3.2 [2.8 - 3.7] |
| T-bil | 0.6 [0.4 - 1] | 0.8 [0.5 - 1.4] | 0.5 [0.4 - 0.8] | 0.7 [0.4 - 1.2] |
| CK | 439 [124 - 1239] | 653 [234 - 2163] | 344 [122 - 1079] | 525 [174 - 1560] |
| CRP | 1.4 [0.3 - 5.3] | 4.7 [1.7 - 12.8] | 0.9 [0.1 - 3] | 1.6 [0.4 - 6.7] |
| SOFA score | 4 [2.3 - 6] | 6 [4 - 8] | 3.8 [2 - 5.2] | 7 [4.9 - 10] |
| 5A score | 3 [3 - 4] | 5 [4 - 6] | 3 [2 - 4] | 5 [4 - 5] |

ADL: Activity of daily living, BT: Body temperature, SBP: Systolic blood pressure, GCS: Glasgow coma scale, WBC: white blood cell count, Hgb: hemoglobin, Hct: hematocrit, PLT: platelet count, BUN: Blood urea nitrogen, TP: Total protein, Alb: serum albumin, T-bil: Total bilirubin, CK: creatine kinase, SOFA: sequential organ failure assessment score

**Supplementary Table 3.** **Hyperparameters in machine learning models**

| **Model** | **Hyperparameter** | **Values** |
| --- | --- | --- |
| Lasso | Minimal lamda | 0.02053 |
| Random forest | Splitting rule | extratrees |
|  | Number of trees | 500 |
|  | Number of Randomly Selected Predictors (mtry) | 30 |
|  | Minimal node size | 1 |
| Gradient boosting tree | Number of Boosting Iterations (nrounds) | 100 |
|  | Shrinkage (eta) | 0.3 |
|  | Max tree depth | 1 |
|  | Minimum Loss Reduction (gamma) | 0 |
|  | Subsample Ratio of Columns (col sample by tree) | 0.6 |
|  | Minimum Sum of Instance Weight (min child weight) | 1 |
|  | Subsample Percentage | 1 |

Please refer to the references for these values.^10-12^

**Supplementary Table 4. Difference of C-statistics in each model**

| **Model** | **C-statistics [95%CI]** | **P-value** |
| --- | --- | --- |
| Lasso | 0.784 [0.717-0.851 | 0.488 |
| Random forest | 0.794 [0.735-0.853] | 0.147 |
| Gradient boosting tree | 0.780 [0.714-0.847] | 0.382 |
| SOFA | 0.787 [0.722-0.851] | 0.419 |
| 5A score | 0.750 [0.681-0.820] | Reference |

The C-statistics were compared to 5A score by Delong test. ^13^

**Reference**

1. Matsuyama T, Morita S, Ehara N, et al. Characteristics and outcomes of accidental hypothermia in Japan: the J-Point registry. *Emerg Med J* 2018;35(11):659-66. doi: 10.1136/emermed-2017-207238 [published Online First: 2018/06/11]

2. Ministry of Health LaW. Ministry of Health, Labour and Welfare website [Available from: <https://www.mhlw.go.jp/index.html>.

3. Okada Y, Matsuyama T, Morita S, et al. The development and validation of a "5A" severity scale for predicting in-hospital mortality after accidental hypothermia from J-point registry data. *J Intensive Care* 2019;7:27.

4. Cowley NJ, Owen A, Bion JF. Interpreting arterial blood gas results. *BMJ : British Medical Journal* 2013;346:f16. doi: 10.1136/bmj.f16

5. Tukey JW. Exploratory data analysis: Reading, MA 1977.

6. Okada Y, Matsuyama T, Morita S, et al. The development and validation of a “5A” severity scale for predicting in-hospital mortality after accidental hypothermia from J-point registry data. *Journal of intensive care* 2019;7(1):27.

7. Fitzgerald M, Saville BR, Lewis RJ. Decision curve analysis. *Jama* 2015;313(4):409-10. doi: 10.1001/jama.2015.37 [published Online First: 2015/01/28]

8. Vickers AJ, Van Calster B, Steyerberg EW. Net benefit approaches to the evaluation of prediction models, molecular markers, and diagnostic tests. *Bmj* 2016;352:i6. doi: 10.1136/bmj.i6 [published Online First: 2016/01/27]

9. Vickers AJ, van Calster B, Steyerberg EW. A simple, step-by-step guide to interpreting decision curve analysis. *Diagnostic and Prognostic Research* 2019;3(1):18. doi: 10.1186/s41512-019-0064-7

10. Package ‘caret’ [Available from: <https://cran.r-project.org/web/packages/caret/caret.pdf> accessed Oct, 22th 2020.

11. Package ‘ranger’ [Available from: <https://cran.r-project.org/web/packages/ranger/ranger.pdf> accessed Oct, 22th 2020.

12. Package ‘glmnet’ [Available from: <https://cran.r-project.org/web/packages/glmnet/glmnet.pdf> accessed Oct, 22th 2020.

13. Package ‘pROC’ [Available from: <https://cran.r-project.org/web/packages/pROC/pROC.pdf> accessed Oct, 22th 2020.
